# Frequency of Translocation t(11;22)(q24;q12) Using Fluorescence In Situ Hybridization (FISH) in Histologically and Immunohistochemically Diagnosed Cases of Ewing’s Sarcoma

**DOI:** 10.7759/cureus.9885

**Published:** 2020-08-20

**Authors:** Muhammad Rizwan Bashir, Shahid Pervez, Atif Ali Hashmi, Muhammad Irfan

**Affiliations:** 1 Pathology, Dallah Hospital, Riyadh, SAU; 2 Pathology and Laboratory Medicine, Aga Khan University Hospital, Karachi, PAK; 3 Pathology and Laboratory Medicine, Intermountain Healthcare, Salt Lake City, USA; 4 Pathology, Liaquat National Hospital and Medical College, Karachi, PAK; 5 Statistics, Liaquat National Hospital and Medical College, Karachi, PAK

**Keywords:** ewing’s sarcoma, fluorescence in situ hybridization (fish), small round blue cell tumors, translocation

## Abstract

Introduction

Ewing sarcoma (ES) family of tumors is one of the most common groups of malignancies arising in children, adolescents, and young adults. Although characteristic histology with immunohistochemical expression of CD99 and FLI1 after exclusion of other small round blue cell tumors is considered diagnostic of ES, frequency of typical ES translocation, i.e., t(11;22)(q24;q12) is not known in our population. Therefore, in this study, we aimed to evaluate the frequency of this translocation in histologically and immunohistochemically diagnosed cases of ES along with its association with other pathological parameters.

Methods

A total of 43 morphologically and immunohistochemically diagnosed cases of ES were included in the study. Fluorescence in situ hybridization (FISH) was performed on representative paraffin blocks to identify t(11;22)(q24;q12) translocation. Association with various clinicopathological characteristics was determined.

Results

Mean age of the patients was 18.23±9.57 years. Bone was the most commonly involved site (22; 51.2%) followed by soft tissue (17; 39.5%) and parenchymal organs (4; 9.3%). A total of 88.4% of cases were found to be FISH-positive for t(11;22)(q24;q12). No significant association of translocation positive cases was noted with tumor size or disease-free survival. Similarly, no significant association of tumor size with disease-free survival was found.

Conclusions

A significant proportion of cases of histologically diagnosed cases of ES exhibited characteristic t(11;22)(q24;q12). This signifies that histology along with immunohistochemistry is reliable for the diagnosis of this tumor; however, in difficult cases, FISH can be performed to detect characteristic translocation. Moreover, we did not find tumor size to be a significant prognostic indicator of survival in ES.

## Introduction

Ewing’s sarcoma (ES), also referred to as primitive neuroectodermal tumor, is one of the most common mesenchymal tumors in children and young adults that can occur in both bones and soft tissues [1]. The tumor was named after James Ewing, an American pathologist, who first described this disease in 1921 [2]. ES accounts for approximately 6% to 10% of malignant bone tumors and is the second most common malignant bone tumor of children and young adults. Histologically, the tumor is uniformly composed of sheets of closely packed small round cells. Glycogen granules are demonstrated in the cytoplasm by periodic acid-Schiff (PAS) stain. Immunohistochemically, ES are positive for vimentin, MIC-2 gene product (CD99), and FLI1. The differential diagnosis of ES is wide, including malignant lymphoma, especially lymphoblastic lymphoma, neuroblastoma, rhabdomyosarcoma, desmoplastic small round cell tumor, and small cell osteosarcoma [3]. However, the diagnosis is reached in most cases only with the help of immunohistochemistry (IHC). On the other hand, some cases require molecular studies for the confirmation of the diagnosis. More than 90% ES cases have the t(11;22) chromosomal translocation. The identification of the highly specific balanced chromosomal rearrangement t(11;22)(q24;q12) in most ESs provides a valuable tool for diagnosing this tumor at the molecular level [4,5].

Chromosomal analysis of ES has advanced further with the development of fluorescence in situ hybridization (FISH) that can be applied to formalin-fixed paraffin-embedded tumor tissue, sparing the need of fresh frozen tissue. Moreover, FISH is found to be more sensitive and specific compared to reverse transcriptase-polymerase chain reaction (RT-PCR) on formalin-embedded tissue sections [6]. In resource-limited countries like Pakistan, FISH cannot be performed on every suspected case of ES for diagnostic confirmation, and we do not even know the frequency of this translocation in our population. Therefore, in this study, we aimed to evaluate the frequency of t(11;22)(q24;q12) in histologically and immunohistochemically diagnosed cases of ES.

## Materials and methods

**Figure 1:**
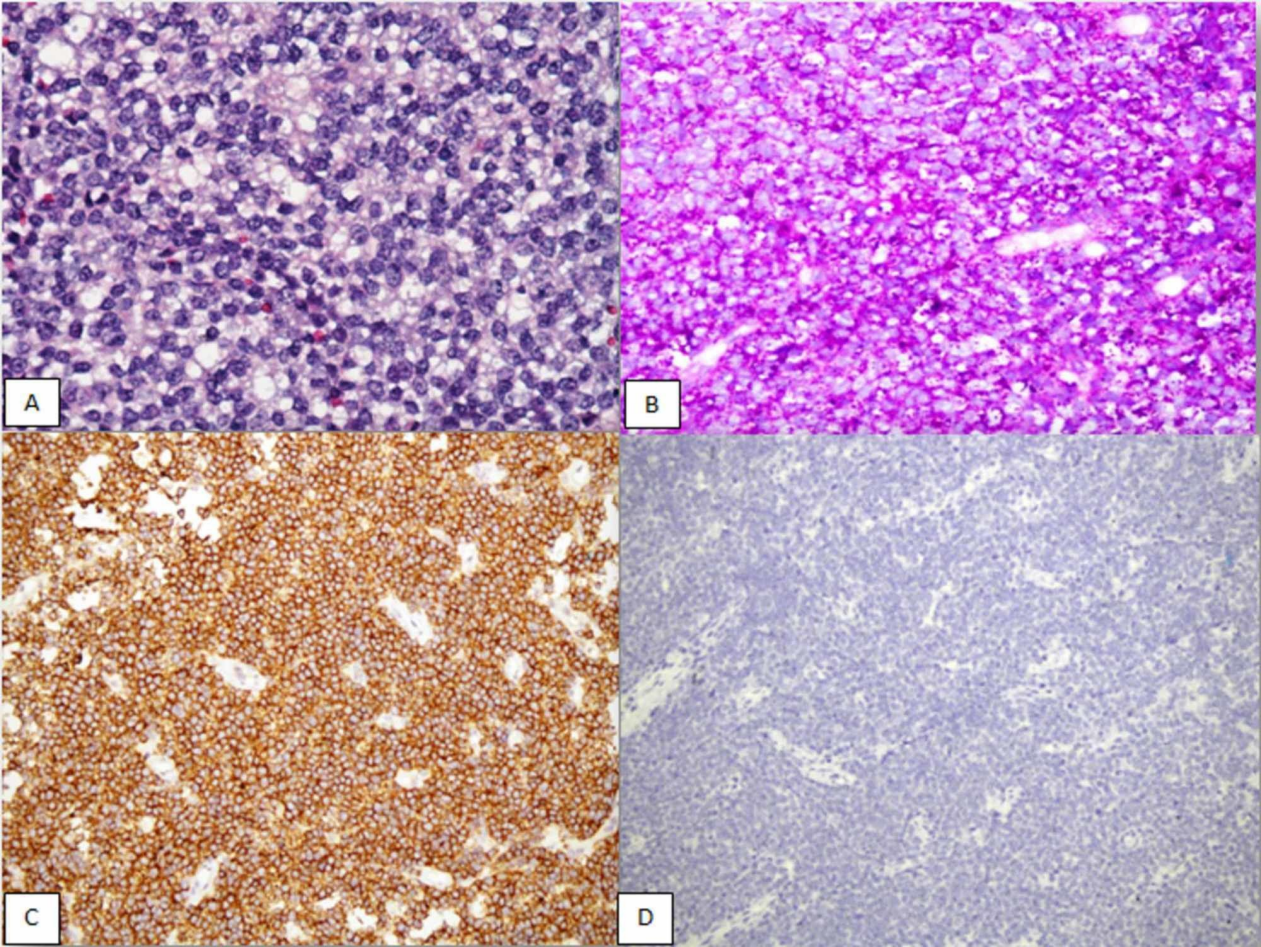
(A) H&E section on 400X showing small round blue cell tumor. (B) Tumor cells showing cytoplasmic glycogen positivity on PAS stain. (C) Tumor cells showing CD99 immunopositivity. (D) Tumor cells negative with desmin immunostain. H&E, hematoxylin and eosin; PAS, periodic acid-Schiff

This is a retrospective observational study conducted at Aga Khan University Hospital’s Histopathology Department over a period of two years, involving 43 morphologically and immunohistochemically diagnosed cases of ES. All cases were excision specimens. Cases with suggestive but not conclusive diagnosis of ES and specimens sent without formalin (without proper fixation leading to loss of cellular details) were excluded from the study. The specimens were macroscopically examined according to the established guidelines. The sections were stained with hematoxylin and eosin (H&E) and PAS, along with a panel of immunohistochemical stains including vimentin, MIC2 (CD99), LCA, TdT, synaptophysin, FLI1, CKAE1/AE3, myogenin, and desmin, as shown in Figure 1.

**Figure 2:**
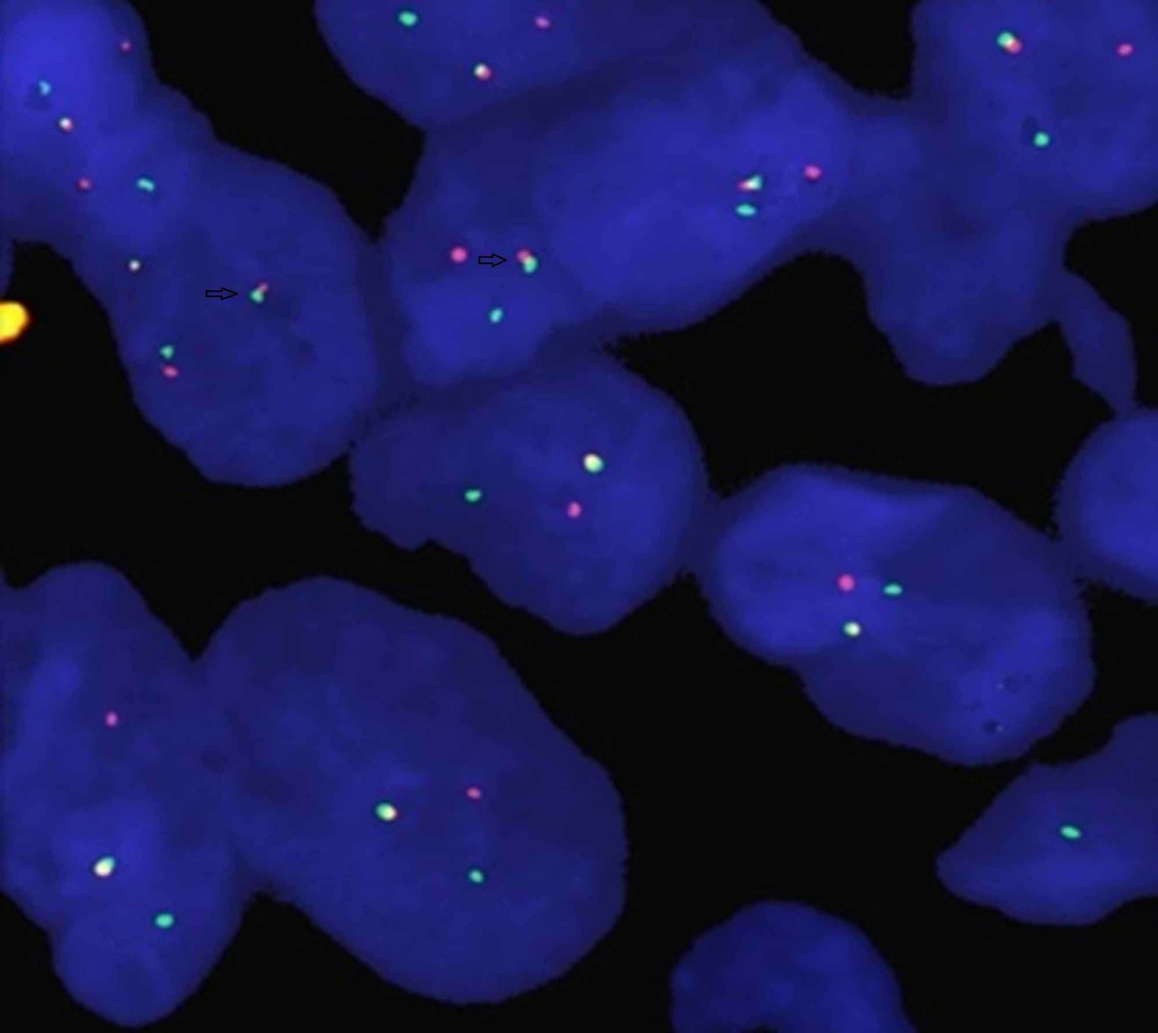
Photograph of one of our positive cases for t(11;22)(q24;q12). Fusion signals (fused green and red signals) are seen in tumor cells (indicated by arrow).

FISH was performed on representative paraffin blocks using LSI EWSR1 Dual Color Break Apart Rearrangement Probe (Vysis, Abbott, Abbott Park, IL, USA) that consists of a mixture of two FISH DNA probes were used for gene amplification. For FISH studies, 4-um-thick sections of tumor tissue taken on coated slides were used, and after deparaffination, the slides were treated with protease. Denaturation of DNA was carried out by immersing the slides in denaturing solution (70% formamide) at 72 degree centigrade for 5 minutes. For hybridization, 10 uL of probe mixture was applied on the target area, and the slide was incubated at 37 degree centigrade overnight. After post-hybridization washes, 10 uL of DAPI (4′,6-diamidino-2-phenylindole) counterstain was applied and a cover slip was placed. The next day, the cover slip was removed, and the slide washed was in a salt/detergent solution to remove any of the probes that should not bind to chromosomes. A differently colored fluorescent dye was added to the slide to stain all of the chromosomes so that they may then be viewed using a fluorescent light microscope. In a normal cell that lacks t(11;22)(q24;q12) in the EWSR1 gene region, a two fusion signal pattern is observed owing to two intact copies of EWSR1. In positive cases with t(11;22)(q24;q12), one fusion and two separate signals (one green and one red) as a result of breakage pattern were seen (Figure 2).

The disease-free survival was determined by reviewing hospital records.

Statistical Analysis

Data were analyzed using Statistical Package For Social Sciences (SPSS) Version 25 IBM Corp., Armonk, NY, USA). Mean and standard deviation were computed for quantitative variables, and frequency and percentage were calculated for qualitative variables. Fisher’s exact test was applied to check association. Kaplan-Meier test was also applied for disease-free survival analysis. P-values ≤ 0.05 were considered as significant.

## Results

**Figure 3:**
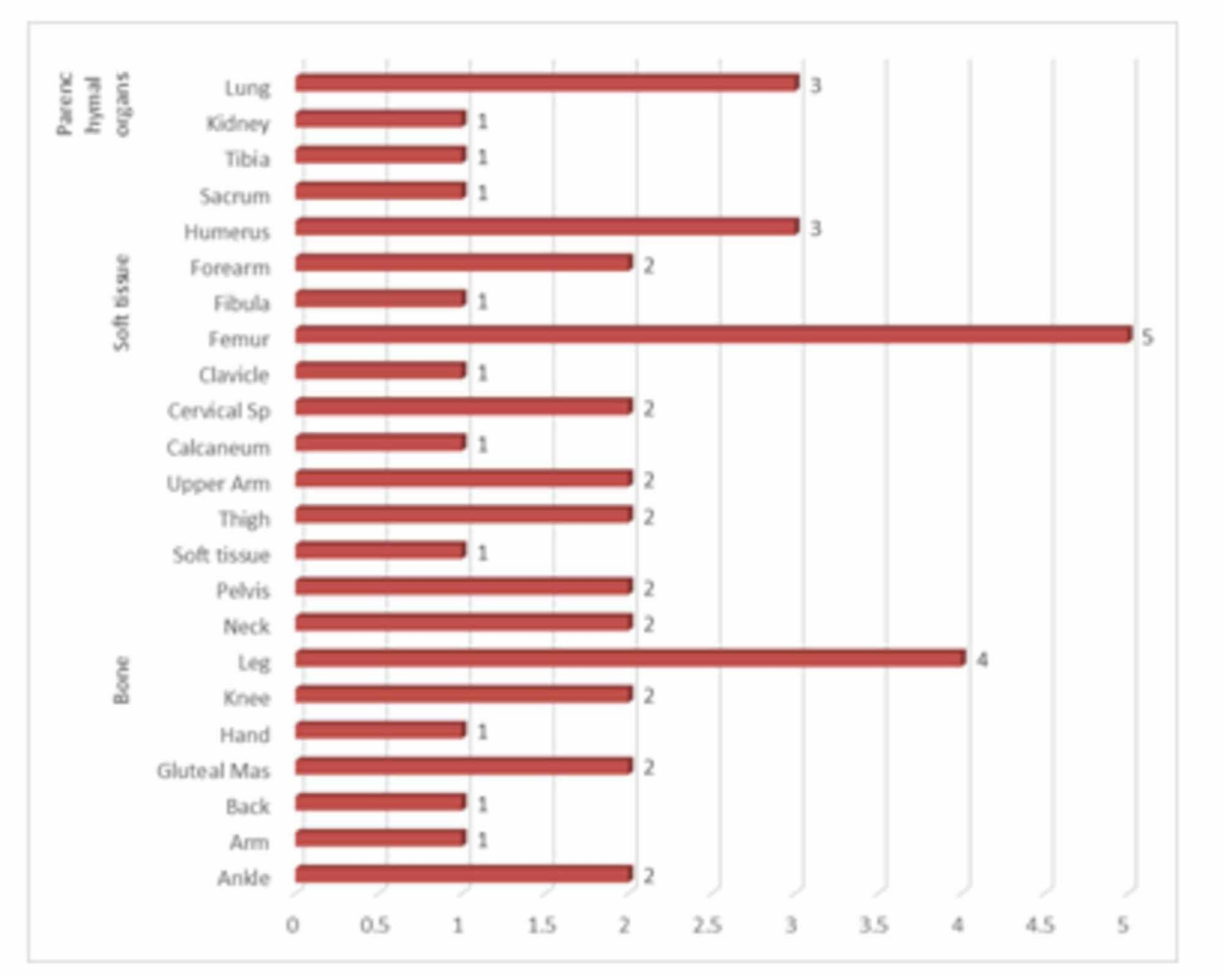
Distribution of sites of involvement of Ewing’s sarcoma

There were 30 (69.8%) male patients and 13 (30.2%) female patients with a mean age of 18.23±9.57 years. Most of the patients were less than 18 years of age. Mean tumor size and disease-free survival were 7.48±4.36 cm and 48.11±31.08 months, respectively. Bone was the most commonly involved site (22; 51.2%) followed by soft tissue (17; 39.5%) and parenchymal organs (4; 9.3%), as shown in Figure 3.

In our study, 88.4% of patients were found to be FISH-positive for t(11;22)(q24;q12). Detailed characteristics of patients are shown in Table 1.

**Table 1:** Clinicopathological characteristics of the study population SD, standard deviation; FISH, fluorescence in situ hybridization

Characteristic	N (%)
Age (years)	
Mean±SD	18.23±9.57
Groups	
<18 years	22 (51.2)
18-35 years	19 (44.2)
>35 years	2 (4.7)
Tumor size (cm)	
Mean±SD	7.48±4.36
Groups	
≤3 cm	8 (18.6)
3.1-8 cm	19 (44.2)
>8 cm	16 (37.2)
Disease-free survival (months)	
Mean±SD	48.11±31.08
Groups	
≤12 months	4 (9.3)
13-48 months	18 (41.9)
>48 months	21 (48.8)
Gender	
Male	30 (69.8)
Female	13 (30.2)
Site	
Bone	22 (51.2)
Soft tissue	17 (39.5)
Parenchymal organs	4 (9.3)
FISH for t(11;22)(q24;q12)	
Positive	38 (88.4)
Negative	5 (11.6)

We found no significant association of FISH-positive t(11;22)(q24;q12) with gender (p=0.0301), age group (p=0.261), tumor size (p=0.580), disease-free survival (p=0.103) and site (p=1.000), as presented in Table 2.

**Table 2:** Association of translocation t(11;22)(q24;q12) positivity with clinicopathological characteristics FISH, fluorescence in situ hybridization Fisher exact test was applied. P ≤ 0.05 was considered as significant.

		FISH, n (%)		p-Value
Characteristic		Positive	Negative	
Gender	Male	25 (65.8)	5 (100)	0.301
	Female	13 (34.2)	0 (0)	
Age group	<18 years	20 (52.6)	2 (40)	0.261
	18-35 years	17 (44.7)	2 (40)	
	>35 years	1 (2.6)	1 (20)	
Tumor size	≤3 cm	8 (21.1)	0 (0)	0.580
	3.1-8 cm	17 (44.7)	2 (40)	
	>8 cm	13 (34.2)	3 (60)	
Disease-free survival	≤12 months	2 (5.3)	2 (40)	0.103
	13-48 months	17 (44.7)	1 (20)	
	>48 months	19 (50)	2 (40)	
Site	Bone	19 (50)	3 (60)	1.000
	Soft tissue	15 (39.5)	2 (40)	
	Parenchymal organs	4 (10.5)	0 (0)	

**Figure 4:**
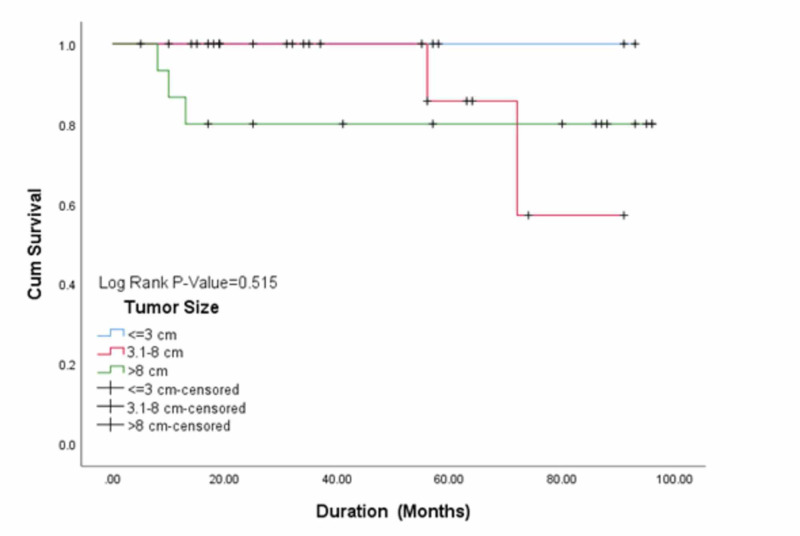
Association of tumor size with disease-free survival

Similarly, no significant association of tumor size with disease-free survival was seen as shown in Figure 4.

## Discussion

In this study, we found a significant proportion of cases of ES to harbor characteristic translocation t(11;22)(q24;q12). On the other hand, the presence or absence of this translocation does not seem to impact the prognosis. Moreover, we did not find any significant association of tumor size with disease-free survival in our patients.

Correctly identifying and diagnosing small round blue cell tumors is a pathological dilemma and specific diagnosis sometimes become very difficult [7,8]. Differential diagnosis largely depends on age and site. For instance, if the location is bone, especially near knee, then small cell osteosarcoma becomes the most important tumor to rule out. On the other hand, in the retroperitoneal location, neuroblastoma and desmoplastic small round cell tumor are the most important differentials. Similarly in adults, Merkel cell carcinoma and metastatic neuroendocrine carcinoma become the important differential diagnosis. Embryonal rhabdomyosarcoma remains the most important differential diagnosis at all sites. IHC is the most useful tool to narrow down differentials in these circumstances [9]. We only included those cases of ES in our study that have unequivocal IHC profile, i.e., all tumors were positive with CD99 and FLI1, some show focal synaptophysin immunoreactivity, and all were negative with CKAE1/AE3, desmin, myogenin, LCA, and TdT immunostains. Still 12% of these cases lacked translocation t(11;22)(q24;q12). This represents the presence of some variant translocations that involves the EWSR1 gene on 22q12 chromosomal region, such as t(21;22), (q22;q12), t(7;22)(p22;q12), or t(2;22)(q33;q12), that result in different fusion products, such as EWSR1-ERG, EWSR1-ETV1, or EWSR1-FEV, respectively [10]. On the other hand, whether these cases represents recently defined Ewing’s-like sarcoma tumors cannot be excluded with confirmation without further molecular analysis [11].

We view our study with a few limitations. Most importantly, we did not investigate the frequency of variant translocations involving the EWSR1 gene, which we think may be present in the remaining 12% of our cases that lacked the translocation. Moreover, FISH break-apart probe cannot differentiate between different EWSR1-FLI1 fusion proteins, i.e., type 1 and type 2, which have shown to have some prognostic significance in different studies [12,13].

## Conclusions

We found 88% frequency of characteristic ES translocation, i.e., t(11;22)(q24;q12), in our study, and this signifies the role of molecular studies in cases difficult to diagnose on routine histopathology and IHC. On the other hand, we did not find any prognostic role associated with the presence of this translocation. Finally, no prognostic significance of tumor size was seen in our cases.
